# Predicting gene ontology functions from protein's regional surface structures

**DOI:** 10.1186/1471-2105-8-475

**Published:** 2007-12-11

**Authors:** Zhi-Ping Liu, Ling-Yun Wu, Yong Wang, Luonan Chen, Xiang-Sun Zhang

**Affiliations:** 1 Academy of Mathematics and Systems Science, Chinese Academy of Sciences, Beijing 100080, China; 2 Graduate University of Chinese Academy of Sciences, Beijing 100049, China; 3 Department of Electronics, Information and Communication Engineering, Osaka Sangyo University, Osaka 574-8530, Japan; 4 Institute of Systems Biology, Shanghai University, Shanghai 200444, China; 5 ERATO Aihara Complexity Modelling Project, JST, Tokyo 151-0064, Japan; 6 Institute of Industrial Science, The University of Tokyo, Tokyo 153-8505, Japan

## Abstract

**Background:**

Annotation of protein functions is an important task in the post-genomic era. Most early approaches for this task exploit only the sequence or global structure information. However, protein surfaces are believed to be crucial to protein functions because they are the main interfaces to facilitate biological interactions. Recently, several databases related to structural surfaces, such as pockets and cavities, have been constructed with a comprehensive library of identified surface structures. For example, CASTp provides identification and measurements of surface accessible pockets as well as interior inaccessible cavities.

**Results:**

A novel method was proposed to predict the Gene Ontology (GO) functions of proteins from the pocket similarity network, which is constructed according to the structure similarities of pockets. The statistics of the networks were presented to explore the relationship between the similar pockets and GO functions of proteins. Cross-validation experiments were conducted to evaluate the performance of the proposed method. Results and codes are available at: .

**Conclusion:**

The computational results demonstrate that the proposed method based on the pocket similarity network is effective and efficient for predicting GO functions of proteins in terms of both computational complexity and prediction accuracy. The proposed method revealed strong relationship between small surface patterns (or pockets) and GO functions, which can be further used to identify active sites or functional motifs. The high quality performance of the prediction method together with the statistics also indicates that pockets play essential roles in biological interactions or the GO functions. Moreover, in addition to pockets, the proposed network framework can also be used for adopting other protein spatial surface patterns to predict the protein functions.

## Background

It becomes an increasingly important task to annotate protein functions when more and more protein sequences and three dimensional structures become available [1]. A fundamental axiom of biology is the cascade that an amino-acid sequence determines a protein structure, and in turn a protein structure determines protein function [2]. Traditionally, functional relationships among proteins are inferred based on the similarities of conserved sequences. However, sequence-based methods have their limits for those proteins with similar structures but distant sequences, in particular they are generally unable to detect protein similarities with convergence evolution or distinguish distant relationships with divergence evolution [3]. High-throughput technologies on structural genomics have produced a large amount of three dimensional protein structure data, which provide valuable complementary information for analyzing ancient relatives situation such as the case that protein folds remain similar after all traces of sequence similarity disappear during evolution [4-7]. However, similarity measure of primary backbone or global folding analysis also fails sometimes, especially when only overall structure information is considered [8,6]. Therefore, methods purely relying on sequence and/or global structure comparison may lead to inaccurate function-related annotation in cases where few residues are responsible for the specificity of functions [9,10].

It is well known that protein functions are mostly determined by physical, chemical and geometric properties of protein surfaces [11], because the surfaces are places where proteins interact with other biological molecules, protein binding and catalytic activities take place. Surface patterns and local spatial distribution of residues are the key to facilitate the function of a protein although they seem to be unrelated with total sequence and global structure of the protein [12-15]. Structural information of protein surface regions enables detailed studies of relationship between a protein structure and its function [16-18]. Identifying similar surfaces between proteins can be useful for understanding biological roles and annotating protein functions. So far there are a number of studies on the computational analysis of protein surface, such as SURFNET [19], LIGSITE [20], CASTp [13,21], eF-Site [22], Cavbase [11], PINTS [23], SURFACE [9], Q-SiteFinder [24], and SCREEN [25]. Some of them have been already used to build comprehensive databases of the identified surface structures.

In this paper, to develop a new method to predict protein functions, we aim to exploit the surface structure information by constructing a pocket similarity network. We choose the CASTp [21] database as our initial source of protein surface patterns. CASTp provides identification and measurements of surface accessible pockets as well as interior inaccessible cavities. In CASTp, a pocket, which is a local spatial surface pattern, is regarded as an empty concavity on a protein surface into which solvent can gain access. The pockets are obtained by a geometric computation method, which can capture the physicochemical texture and the shape of a surface around functional residues, from the protein structures in PDB [26]. As geometrically defined surface patterns, the pockets are believed to have rather strongly clue to protein functions [27,28], and thereby are adopted as basic elements to construct the structure similarity network in this paper.

For the protein functions, we focus on the prediction of Gene Ontology (GO) terms [29]. GO is a functional classification system of gene products and was developed to address the need for consistent descriptions of gene products in different databases. GO organizes biological terms assigned to one of the three controlled vocabularies (ontologies): molecular functions, biological processes and cellular components. GO also includes relationships between terms such as specialization or past-whole relations. GOA is a database to provide high-quality GO annotations to proteins [30]. In this paper, the GO terms of a protein is predicted, based on the pocket similarity network constituted from a set of annotated proteins in GOA database. A pocket similarity network is a network with its nodes representing pockets and edges indicating the similarities of each pair of pockets. We assume that the functions of a protein are mainly determined by the pockets it contains. If two pockets are similar enough, the corresponding proteins maybe share some common functions. In other words, if one pocket in a protein is similar to many pockets in different proteins which share some GO terms, the protein is related to the same GO terms with a high probability. Based on the pocket similarity network, the proposed procedure of the prediction is implemented in the following way. That is, every protein is simply considered as a set of pockets, and the GO terms annotated to a protein are attributed to each of its pockets. Then based on the correspondence scores between pockets and GO terms in the annotated proteins, the scores between a new protein and GO terms are obtained by comparing all pockets of this new protein with other pockets.

Cross-validation experiments are conducted to evaluate the performance of the proposed method. Numerical results are demonstrated in the recall-precision graphs, which verify high effectiveness of the proposed method for both prediction accuracy and computation efficiency. Such computational results together with statistical analysis also reveal that similar surface pockets in proteins are essential to biological activities and are strong clues to similar GO functions. In contrast to the existing approaches which are mainly suitable for homologous proteins, the proposed method is not only effective for the proteins with distant relationships or with convergent evolution, but also can further reveal direct links between small surface patterns and GO functions, which actually can be explored to identify active sites or functional motifs.

## Results

### Data Sets

As well known, there are many redundant (due to different experiments) or similar (due to same protein family) structures in PDB, which result in biased statistics. In order to eliminate the unexpected side effects, the PDB_SELECT 25 [31] was chosen in this study. The PDB_SELECT database is a subset of the structures in the PDB that does not contain highly homologous sequences. In the PDB_SELECT 25, no two proteins have more than 25% sequence identity for alignments of length 80 or more residues. Note that PDB_SELECT 25 only contains individual chains of proteins, while all chains of each protein are used in the experiments.

For each protein in the dataset, we retrieved all of its pockets from CASTp [21] when available. Then the surface pocket similarity network was constructed according to the given parameters (see Methods for details). Each node in the network represents a pocket. The edge between two nodes means that the similarity of the corresponding two pockets are beyond a given threshold.

The similarity of two pockets was evaluated by pvSOAR server [28]. An example of searching results is illustrated in Additional File 1. Some proteins that not covered by pvSOAR database are discarded. According to the different similarity thresholds, several pocket similarity networks were obtained and used in the experiments. Obviously, the tighter the threshold is, the less the edges in the network are. Some nodes become isolated when the threshold is tight enough, which means that no similar pocket can be found for the given threshold. The isolated nodes were deleted from the network since they are useless for the following study. An example of the pocket similarity network with detail descriptions is also given in Additional File 1.

In the pocket similarity network, a node or pocket is called *GO annotated node *if the pocket is from a GO annotated protein. Similarly, an edge is called *GO annotated edge *if two endpoints of the edge are both annotated. An annotated edge is called a *GO related edge *if two endpoints of the edge are annotated by at least one common GO term.

All data used in the experiments were retrieved from the related websites, and the versions of databases are as follows: CASTp (2006-01-20), pvSOAR (2006-07-13), GO (2006-09-01) and GOA (2006-05-31).

### Statistics Results

In order to explore the relationship between the pocket similarity and the GO terms similarity, we calculated the percentage of the GO related edges, i.e., the percentage of the pocket pairs of which the two corresponding proteins have at least one common GO term. The results in several pocket similarity networks constructed by different thresholds are listed in Tables 1, 2, 3, 4. Table 1 shows the results by using the E-value of sequence similarity as the threshold. Table 2 shows the results by using the p-value of structure cRMSD similarity as the threshold. Table 3 is the results by taking the p-value of structure oRMSD similarity as the threshold. Table 4 uses the combination of E-value of sequence similarity and p-value of cRMSD similarity, i.e. both the E-value and p-value are required to satisfy the given threshold. The cRMSD is calculated by original coordinates of atoms, and the oRMSD is an alternative measure of dissimilarity which is developed based on the unit vector RMSD. Namely, the method first projects each residue onto the unit sphere and then calculate the discrimination by RMSD [27,21].

**Table 1:** The percentage of the edges (pocket pairs) related to similar GO terms in pocket similarity network which is constructed by using the E-value of sequence similarity between two pockets as the threshold.

Threshold	1.0 × 10^-1^	1.0 × 10^-2^	1.0 × 10^-3^	1.0 × 10^-4^	1.0 × 10^-5^
Pocket pairs	3178	1460	652	320	219
GO annotated pairs	2359	1086	492	241	160
Similar pairs	581	375	252	178	126

Percentage	24.63%	34.53%	51.21%	73.85%	78.75%

**Table 2:** The percentage of the edges (pocket pairs) related to similar GO terms in pocket similarity network which is constructed by using the p-value of structure cRMSD similarity between two pockets as the threshold.

Threshold	1.0 × 10^-1^	1.0 × 10^-2^	1.0 × 10^-3^	1.0 × 10^-4^	1.0 × 10^-5^
Pocket pairs	1002	602	521	481	468
GO annotated pairs	778	501	437	405	397
Similar pairs	508	464	430	403	396

Percentage	65.29%	92.61%	98.40%	99.50%	99.75%

**Table 3:** The percentage of the edges (pocket pairs) related to similar GO terms in pocket similarity network which is constructed by using the p-value of structure oRMSD similarity between two pockets as the threshold.

Threshold	1.0 × 10^-1^	1.0 × 10^-2^	1.0 × 10^-3^	1.0 × 10^-4^	1.0 × 10^-5^
Pocket pairs	2354	1182	757	618	567
GO annotated pairs	1786	922	617	516	483
Similar pairs	626	564	515	491	475

Percentage	35.05%	61.17%	83.47%	95.16%	98.34%

**Table 4:** The percentage of the edges (pocket pairs) related to similar GO terms in pocket similarity network which is constructed by using the combination of E-value of sequence similarity and p-value of structure cRMSD similarity between two pockets as the threshold.

Threshold	1.0 × 10^-1^	1.0 × 10^-2^	1.0 × 10^-3^	1.0 × 10^-4^	1.0 × 10^-5^
Pocket pairs	711	360	257	189	145
GO annotated pairs	551	293	203	148	109
Similar pairs	402	287	203	148	109

Percentage	72.95%	97.95%	100%	100%	100%

In these tables, the first row gives the similarity thresholds, which are ranged from 1.0 × 10^-1 ^(loosest) to 1.0 × 10^-5 ^(tightest) from left to right. The second row indicates the numbers of pocket pairs that satisfy the given threshold, i.e. the numbers of edges in the pocket similarity network. The third row lists the GO annotated edges, i.e. the pocket pairs which are both from GO annotated proteins. Some of the edges are not GO annotated edges, and therefore removed in the next experiments. The fourth row shows how many GO annotated edges in the given pocket similarity network are GO related, i.e. the corresponding proteins of such an edge have at least one identical GO function. The last row is the percentages of GO related edges, which are obtained by dividing the corresponding numbers in the fourth row by the numbers in the third row. The percentage is the statistical significance of the pocket pairs with at least one identical GO function in all GO annotated pocket pairs.

Obviously, when the thresholds become tighter, i.e. two pockets in an edge become more similar, the probability of the corresponding proteins with at least one identical GO term increases. The tendency becomes more significant when the structure similarities such as cRMSD and oRMSD are used instead of the sequence similarity as the threshold, which are illustrated in Tables 2 and 3.

**Figure 1:**
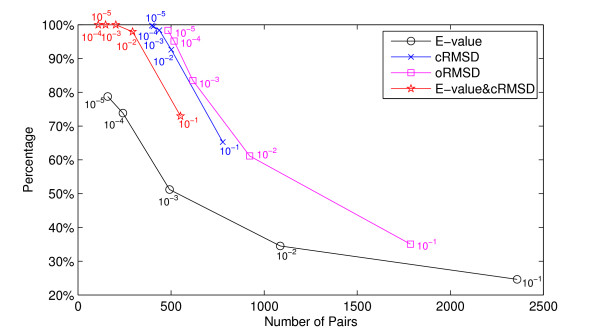
**Similar pockets imply similar functions**. The relationship between pocket similarity and functional similarity. The graph summarizes the results in Tables 1-4. We use four statistical significance measurements as the thresholds to construct the similarity network, that is E-value of the sequence similarity, p-value of structure similarity cRMSD, p-value of structure similarity oRMSD, and the combination of E-value and p-value of cRMSD. The horizontal axis represents the number of GO annotated edges (i.e. pocket pairs) found by given thresholds. The vertical axis denotes the percentage of GO annotated edges with at least one identical GO term between two end nodes. The graph shows that the structure-based threshold is better than the sequence-based threshold.

Figure 1 shows the percentage of GO related edges in the pocket similarity network with different thresholds, compared with the number of GO annotated edges. As shown in Figure 1, with the same number of GO annotated edges, clearly the pocket similarity network using structure similarity has more GO related edges, comparing with the network based on sequences, which provides the evidence that the structure similarity can determine protein functions more precisely than sequence similarity. This is consistent with the common recognition in biology.

We also computed the frequencies of GO functions in the GO related edges. The GO functions in a GO related edge are the common GO functions of two proteins corresponding to the edge. The top 15 functions found from a pocket similarity network (see Additional File 1) are mostly binding and catalytic activity functions, which are consistent with the existing results in the literature that the pockets or clefts on protein surface play important roles, such as binding [32,17].

We also analyzed the relationships between functions annotated to a node and the most frequent GO functions in its closest neighbors. The results (see Additional File 1) show that the more frequently a GO term occurs in the closest neighbors of a pocket, the higher probability of this GO term the protein containing this pocket has. This motivates a straightforward prediction method to predict protein GO functions.

These statistics results give the strong implications that proteins with similar pockets may share some common GO terms and the high correlation between the pocket similarity and GO annotations. The pocket similarity can be used to predict protein functions, even when no global sequence or structure similarity is available. The tendency that more similar pockets would have more similar functions is also a clue, that is, protein surface pockets play key roles in facilitating protein functions.

### Prediction Results

According to the statistics results (see Tables 1, 2, 3, 4), the pocket similarity network constructed by using p-value 10^-2 ^of the structure similarity cRMSD as the threshold was used in prediction experiments. This network consists of 831 nodes and 602 edges, in which 501 edges are annotated. In the network, the pockets belong to 397 proteins from different families, in which 316 proteins are GO annotated.

The leave-one-out cross-validations were conducted to evaluate the performance of the proposed method. That is, in each validation a protein was selected as a target protein from 316 GO annotated proteins. The correspondence scores between each pocket of the target protein and GO terms were calculated from the annotated closest neighbors (not in the target protein) according to the score scheme presented in section Methods. Then the correspondence scores between the target protein and GO terms were inferred based on the scores of its pockets.

**Figure 2:**
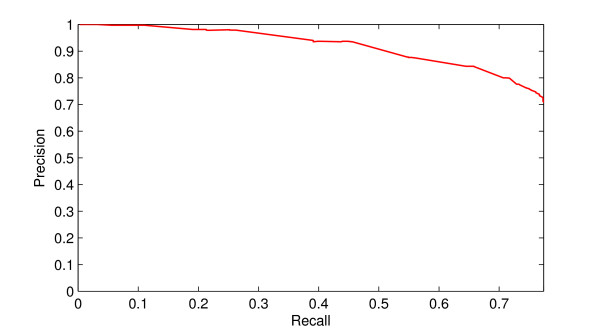
**Recall-precision graph**. Recall-precision relationship graph for the prediction result.

The recall-precision graph of the prediction is shown in Figure 2. To draw the recall-precision graph, we sorted the correspondence scores between every pair of target protein and GO term, and then used these scores as a threshold to filter the original predicted result. Every threshold generates a recall value and a precision value, which is plotted as a point in the recall-precision (RP) graph. The high position of the RP curve shows that presented prediction method is effective. The maximum F-measure is 0.756.

The original (i.e. unfiltered) predicted result has recall value 0.774 and precision value 0.706. In the 316 testing proteins, there are 267 proteins, each of which has at least one GO term predicted in the experiments. The prediction method does not hit any GO term of the rest 49 proteins. The coverage is 84.50%, which is considerably high and quite efficient for predicting protein functions. The result illustrates that the presented method can rather correctly predict GO functions for most proteins, even if they come from different protein families, or there is no homologous information available.

**Figure 3:**
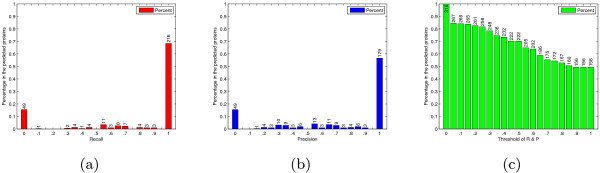
**Statistics of individual predictions**. The distributions of proteins with respect to different (a) recall and (b) precision values. The concrete numbers of proteins are shown on each bar individually. Most of the 316 proteins have high recall and precision values, which mean that their functions are almost covered by prediction results and the false positive is low. (c) is the percentages of proteins with both recall and precisions higher than the given thresholds. 156 proteins can be predicted with recall value 1 and precision value 1.

For the prediction results of individual proteins, most proteins have high recall and precision values as shown in Figure 3. The distribution of 316 proteins with regard to different recall values is drawn in Figure 3(a), while the distribution with regard to precision values is in Figure 3(b). In the 316 GO annotated proteins, 267 proteins have non-zero recall values, i.e. at least one GO term of each protein is predicted correctly. And in these 267 proteins, 216 proteins have recall value 1, which means that all GO terms associated with them are predicted correctly. Considering the precisions, 179 proteins have precision value 1, i.e. all predicted GO terms of these proteins are correct. The percentages of the proteins with both recall and precision higher than the given thresholds are shown in Figure 3(c). There are 156 proteins with both recall 100% and precision 100%. Figure 3 further illustrates the high performance of the presented prediction method in terms of effectiveness and accuracy.

**Figure 4:**
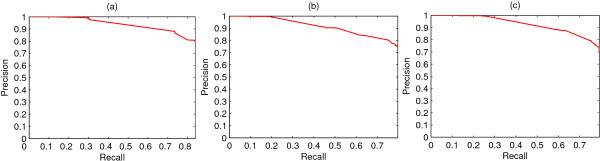
**Recall-precision graphs for the three ontologies**. Recall-precision relationship graph of prediction for the three GO ontologies independently. (a) cellular component ontology, (b) molecular function ontology, (c) biological process ontology.

Since Gene Ontology is composed by three relatively independent ontologies, the prediction results on the three ontologies are summarized respectively. The three recall-precision graphs are shown in Figure 4 and the details are listed in Table 5. These results are similar to the integrated version described above.

**Table 5:** The prediction results of proteins by the three GO ontologies individually. These results are similar to the integrated version, and show that the performance of the proposed prediction method does not heavily depend on the considered GO terms. For Gene Ontologies, C means cellular component, F means molecular function, and P means biological process. The proteins with R & P 100% mean that these proteins can be predicted with recall value 1 and precision value 1.

Gene Ontology	C	F	P
Maximum F-measure	0.823	0.778	0.772
Recall-precision	(0.839, 0.805)	(0.797, 0.729)	(0.792, 0.715)
Number of proteins	92	290	275
Predicted proteins	81	249	233
Not predicted	11	41	42
Proteins with recall 100%	74	207	202
Proteins with precision 100%	69	174	166
Proteins with R & P 100%	66	155	150

We also did computational experiments on the pocket similarity networks featured by different thresholds. The results are similar to those in this section. When the threshold of the pocket similarity network becomes tighter, e.g. from 10^-3 ^to 10^-5^, the recall-precision curve moves to a higher position, and the prediction coverage and accuracy also increase simultaneously. For instance, if the threshold is 10^-3^, the maximum F-measure increases to 0.875, the recall is 0.888 and the precision is 0.839. One obvious disadvantage for a tight threshold is that the number of proteins involved in the pocket similarity network is fewer than that with a loose threshold. Such a tradeoff between performance and scale is shown in Table 6. The other prediction results of the pocket similarity networks by using different p-value of structure similarity cRMSD as the threshold can be found in Additional File 1.

**Table 6:** Prediction results in the pocket similarity network by using different cRMSD p-values as thresholds. The detailed prediction results of thresholds from 10^-3 ^to 10^-5 ^are listed in the Additional Files.

Threshold	10^-2^	10^-3^	10^-4^	10^-5^
Maximum F-measure	0.756	0.875	0.898	0.904
Recall-precision	(0.774,0.706)	(0.888,0.839)	(0.907,0.869)	(0.913,0.877)
Coverage	84.50%	96.98%	98.35%	99.16%
Number of proteins	316	265	242	237

We also predicted the GO terms for the 81 un-annotated proteins and verified the predicted results by the functional information from other sources such as PDB and literature. The predicted results are almost consistent with the existing knowledge. Some results for un-annotated proteins are listed in Table 7 and the full list can be found at our website.

**Table 7:** Some predicted GO terms of the un-annotated proteins. The predicted results are almost consistent with the existing functional knowledge in databases and literature. The full predicted results of all un-annotated proteins in the considered pocket similarity network can be found at our website.

Protein	Predicted GO terms	GO description	Information
1orn	GO:0003677 F	DNA binding	PDB Classification: Hydrolase/dna
	GO:0019104 F	DNA N-glycosylase activity	
	GO:0003906 F	DNA-(apurinic or apyrimidinic site) lyase activity	
1lia	GO:0009503 C	light-harvesting complex (sensu Viridiplantae)	PDB Classification: Light Harvesting Protein
	GO:0015979 P	photosynthesis	PMID: 11134927
1dnl	GO:0010181 F	FMN binding	PDB Classification: Oxidoreductase
	GO:0004733 F	pyridoxamine-phosphate oxidase activity [source: EC:1.4.3.5]	EC no.: 1.4.3.5
1jbe	GO:0004871 F	signal transducer activity	PDB Classification: Signaling Protein
	GO:0000160 P	two-component signal transduction system (phosphorelay)	PMID: 12270703
1uap	GO:0005509 F	calcium ion binding	PDB Classification: Protein Binding
	GO:0004252 F	serine-type endopeptidase activity	PMID: 12670942
1fb3	GO:0016491 F	oxidoreductase activity	PDB Classification: Oxidoreductase
	GO:0004324 F	ferredoxin-NADP+ reductase activity [source: EC:1.18.1.2]	EC no.: 1.18.1.2
	GO:0050660 F	FAD binding	
	GO:0050661 F	NADP binding	
	GO:0006118 P	electron transport	
1got	GO:0005834 C	heterotrimeric G-protein complex	PDB Classification: Complex (gtp Binding/transducer)
	GO:0004871 F	signal transducer activity	PMID: 17036056
	GO:0007186 P	G-protein coupled receptor protein signaling pathway	
1k8g	GO:0003677 F	DNA binding	PDB Classification: DNA Binding Protein/dna
	GO:0042162 F	telomeric DNA binding	PMID: 11743727
	GO:0006260 P	DNA replication	
1iis	GO:0042612 C	MHC class I protein complex	PDB Classification: Immune System
	GO:0030106 F	MHC class I receptor activity	
	GO:0019882 P	antigen processing and presentation	
1lug	GO:0008270 F	zinc ion binding	PDB Classification: Lyase
	GO:0004089 F	carbonate dehydratase activity [source: EC:4.2.1.1]	EC no.: 4.2.1.1
	GO:0006730 P	one-carbon compound metabolic process	Chemical Component: ZINC ION

### Results by Protein Similarity

In order to evaluate and justify the significance of pocket similarity, we constructed the protein similarity network by using a similar method as that used in the construction of the pocket similarity network. The proteins in the same dataset were compared all-against-all using CE algorithm [33]. In the protein similarity network, a node represents a protein, while the edge is created if the CE Z-Score of two linked proteins exceeds a given threshold. Then the leave-one-out prediction tests were conducted in the protein similarity networks with different Z-Score thresholds. The prediction results of the Z-Score thresholds 3.8, 4.8 and 5.8 can be found in Additional File 1, with the statistics of prediction status.

**Figure 5:**
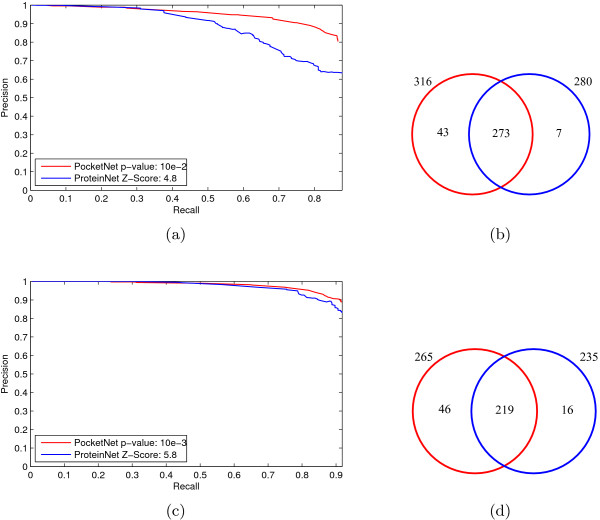
**Comparison of prediction results between methods by global and local similarity**. The recall-precision graphs of the prediction results by the pocket similarity network *versus *those by the protein similarity network in the intersection of proteins. (a) The RP curve of the prediction results by pvSOAR cRMSD p-value 10^-2 ^*versus *CE Z-Score 4.8. (b) The intersection illustration of the proteins that can be predicted by the two methods. The red cycle represents the pocket similarity network and the blue cycle the protein similarity network. The RP graph is drawn in the results of the intersection of two cycles. (c) and (d) are the similar comparison between pvSOAR cRMSD p-value 10^-3 ^and CE Z-Score 5.8.

Figure 5 shows the comparison between the RP curves and corresponding coverage by different thresholds of pocket similarity (pvSOAR cRMSD p-value) and protein similarity (CE Z-Score). The RP curves are drawn in the proteins that both two methods can predict. Figure 5(a) is the comparison of RP graphs between the cRMSD p-value 10^-2 ^and the Z-Score 4.8, while Figure 5(b) is the coverage. Similarly, Figure 5(c) and 5(d) are the comparison between the cRMSD p-value 10^-3 ^and the Z-Score 5.8. Additional comparisons between different cRMSD p-values and Z-Scores can also be found in Additional File 1. There are more proteins, which can only be predicted by pocket similarity, than those which can only be predicted by their global structure similarity. And the prediction quality of pocket-based method is better than that of global-structure-based method in the common proteins. The comparison results between the pocket similarity networks and the protein similarity networks show the obvious improvement of the prediction performance on surface similarity in contrast to global similarity, in terms of both prediction coverage and precision.

### Results by GO Relevance Information

The GO organizes the terms as directed acyclic graphs (DAG), where the child term is more specific and informative than its ancestors. Generic terms do have less relevance for comparing the functional similarity between proteins. Therefore the presence of unspecific GO terms may bias the statistics and prediction results. Two experiments are conducted to evaluate the influence of the unspecific GO terms. In the first experiment, the GO semantic similarity [34-36] of each GO annotated edge is calculated by the relevance similarity proposed in [36], which takes into account both the relevance information of GO terms and the functional similarity between GO terms. The distributions of GO semantic similarity in different pocket similarity networks are illustrated in Additional File 1. Most of the pairs of proteins with similar pockets in the pocket similarity network have significant semantic similarity. This implies that the similar pockets may correspond to similar functions in the semantic similarity measurement.

**Figure 6:**
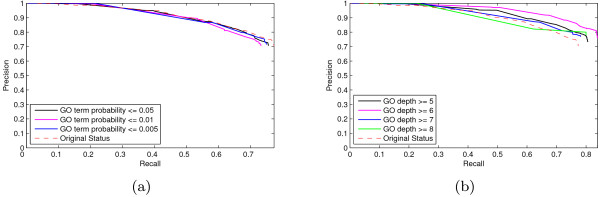
**The prediction results with selected GO terms**. The recall-precision graphs of the prediction results with selected specific GO terms by the pocket similarity network (pvSOAR cRMSD p-value 10^-2^). (a) The RP curves of the prediction results when the informative GO terms are selected based on GO term probability. (b) The RP curves of the prediction results when the informative GO terms are selected based on the depth level. The red dash line gives the original prediction results without filter.

In the second experiment, the unspecific GO terms are discard from the prediction results. The relevance information of GO terms are represented by their probability [34] to occur in a dataset. The frequency of a GO term is defined as

freq(c)=anno(c)+∑h∈children(c)freq(h),
 MathType@MTEF@5@5@+=feaafiart1ev1aaatCvAUfKttLearuWrP9MDH5MBPbIqV92AaeXatLxBI9gBaebbnrfifHhDYfgasaacPC6xNi=xI8qiVKYPFjYdHaVhbbf9v8qqaqFr0xc9vqFj0dXdbba91qpepeI8k8fiI+fsY=rqGqVepae9pg0db9vqaiVgFr0xfr=xfr=xc9adbaqaaeGacaGaaiaabeqaaeqabiWaaaGcbaGaemOzayMaemOCaiNaemyzauMaemyCaeNaeiikaGIaem4yamMaeiykaKIaeyypa0JaemyyaeMaemOBa4MaemOBa4Maem4Ba8MaeiikaGIaem4yamMaeiykaKIaey4kaSYaaabuaeaacqWGMbGzcqWGYbGCcqWGLbqzcqWGXbqCcqGGOaakcqWGObaAcqGGPaqkaSqaaiabdIgaOjabgIGiolabdogaJjabdIgaOjabdMgaPjabdYgaSjabdsgaKjabdkhaYjabdwgaLjabd6gaUjabcIcaOiabdogaJjabcMcaPaqab0GaeyyeIuoakiabcYcaSaaa@5B57@

in which *anno*(*c*) is the number of proteins annotated with the term *c*, and *children*(*c*) is the set of child nodes of term *c*. The probability of the term *c *is then defined as *p*(*c*) = *freq*(*c*) = *freq*(*root*), where *freq*(*root*) is the frequency of the root term [36]. The probability is monotonically increasing when moving up on a path from a leaf to the root. A GO term with smaller probability should be more specific and informative. The probabilities of all GO terms occurring in the dataset are calculated. The mean values of the probabilities in three ontologies are 0.044 (C), 0.020 (P) and 0.015 (F) respectively. Then we discard GO terms with probabilities larger than a given value (from 0.05 to 0.005) in the prediction to reduce the possible bias caused by unspecific GO terms with high frequency. The recall-precision graphs of prediction results with selected GO terms are drawn in Figure 6(a). We also consider the depth levels of GO terms. The recall-precision graphs of prediction results with the GO terms under certain depth level are drawn in Figure 6(b). The results show that the prediction method is effective for specific GO terms and the results are not artificially biased.

## Discussion

The protein surface patterns such as pockets have been shown to be important to protein functions. Some functions of pockets on protein surfaces were already confirmed [32,17]. The statistics in this paper also show that the proteins containing similar pockets may have similar functions. There are many approaches to predict protein functions by global or local structure similarity [23,37]. Most of the existing methods are based on structure and/or sequence alignments. In this paper, we proposed a novel approach, which predicts protein functions based on comparison of predefined local surface patterns instead of using structure alignments to find function-related structure motifs. It is an advantage to use the information of multiple surface patterns instead of annotating precisely protein functions to a single pocket because the proposed prediction method can exploit local surface similarity information. But the information provided by pockets may not be in the same level, for example, some pockets may be more closely related to particular protein functions than others, and such difference is not considered in the current work. The introduction of informative structure motifs such as in [23,37] to the proposed method can also improve the results. In addition, the proposed method can be straightforwardly extended to prediction models with other definitions of locally structure surface patterns as introduced in the first section.

### Scoring Scheme

Although the statistics have shown that proteins with similar pockets have high probabilities to have similar GO functions, we do not assume that a particular pocket determines protein functions when calculating the correspondence scores between pockets and GO terms. In other words, the score is evaluated based on the effect of multiple pockets. The score of a pocket corresponding to a particular GO term is the normalized frequency of the GO term annotated to its closest neighbors. The more frequently the GO term appears in the closest neighbors, the larger the score is. This scoring scheme is very simple and does not explicitly consider the influence of the size of neighborhood. For example, a pocket with many closest neighbors often has smaller scores than the pockets with a very few neighbors. It maybe affect the final prediction results. The scores will also be affected by the total number of GO terms belonging to the neighboring proteins. If some neighboring proteins have many GO functions, the scores of the pocket will be smaller than average. The sensitivity of prediction results to the scoring scheme is important for the application of prediction method, and needs to be further studied in the future. The prediction accuracy may be improved if more elaborated scoring methods are used.

### Properties of Pocket Similarity Networks

In the proposed method, only the information of the closest neighbors are exploited. In fact, we found that the pocket similarity networks have many interesting properties. For example, the pocket similarity networks are almost sparse and have some modularity. We used the pockets in the same connected components to infer the correspondence scores between a pocket and GO functions. The prediction results are very close to those of the closest-neighbor-based method (see Additional File 1). Furthermore, if the edges are dense in each group and sparse between any two groups, the pocket similarity network can be partitioned into several groups (modules), where each group can be regarded as a candidate of a pocket family, i.e. a classification of pockets. Then each pocket family could be annotated with functions and used to predict protein functions. The pocket classification will be a further research topic of our study.

### Sources of Pocket Similarity

The pvSOAR database is an important but not the only source of pocket similarity. For example, we can compare each pair of pockets by structure alignment tools such as DALI [38], CE [33] and SAMO [39], and use the alignment results as an independent or additional threshold. In the future, we will identify tenser similar pockets by this method and detect the physicochemical and geometric characteristics of these functionally important surface motifs. The works on additional pocket similarity information and identification of biochemical features on these spatial motifs are still ongoing and will be presented in another paper.

### GO Annotations

Some proteins are not annotated in current GOA database, and their prediction results are partly shown in Table 7. The results reveal direct links between small structural pockets and biological functions. Such information can be further exploited to identify active sites and functional motifs by combining with other biological datasets. We can also predict the GO functions of un-annotated proteins by using the proposed method, and then use the predicted GO terms to predict other protein with unknown functions, as if they are already annotated. Whether or not this method can really improve the prediction accuracy and coverage needs further study in the future. Another important research direction is to exploit the GO hierarchical structure and the semantic similarity in the prediction method to improve the accuracy [40].

### Detection of functional sites

The main point of this paper is utilizing local structure information to improve the effectiveness and accuracy of protein function prediction. It is also very interesting and important to check those functionally similar proteins which have local structure similarity instead of global one to find the functional sites in detail and identify their functions. Our main concern is current data are far from complete to do this. Comprehensive experiments on complete local structure library and larger structure databases such as whole PDB may be necessary to archive interesting and convincible results for detecting functional sites. Also the prediction accuracy may be further improved. Our future attempts will eventually take into account the detailed local structural properties related to protein function.

## Conclusion

In conclusion, a novel prediction method was proposed in this paper to predict protein GO functions from the surface pocket similarity, by directly linking structural patterns with biological functions. In addition to the high coverage and accuracy of the predictions, the prediction method is also simple and computationally efficient, and therefore can be applied to large-scale problems. The statistics and computational experiments show the effectiveness of the method. It is a supplement to the existing prediction methods based on sequence and/or global structure similarity. In contrast to the existing methods which are mainly effective for homologous proteins in divergent evolution, the method in this paper is also suitable for the proteins with distant relationship or with convergent evolution.

## Methods

### Constructing Pocket Similarity Network

The pocket similarity network is a network in which each node represents a pocket and each edge connects two similar pockets. For each considered protein, all its pockets can be obtained from CASTp database. Then an edge is linked between a pair of nodes, if their similarity measure satisfies the given threshold. The pvSOAR is an all-against-all comparison database of pockets in CASTp. For each similar pocket pair, the pvSOAR database provides three statistical significant measurements: E-value of sequence similarity, p-value of structure similarity cRMSD, p-value of structure similarity oRMSD. These three measurements are explained in [21,28] and the documentation in pvSOAR server. We can select one of these measurements or their combination as the threshold to build the network. In detail, we query each pocket in the pvSOAR database to find the similar pockets which satisfy the given threshold, and then link an edge between the queried pocket and the hitting pocket. After all edges are added, the isolated nodes, i.e. nodes without any linking edge, are removed from the network.

### Scoring Pockets and Proteins for Function Prediction

**Figure 7:**
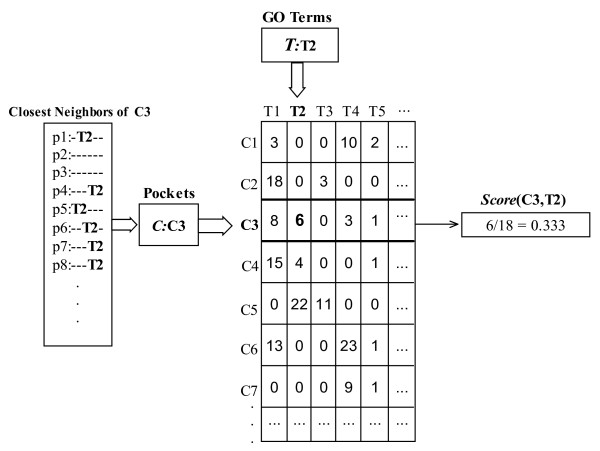
**Example of pocket scoring**. An example of pocket scoring scheme. The closest neighbors of the pocket C3 and the corresponding GO terms are listed in the left box. The occurrence numbers of GO terms in the closest neighbors of pocket C3 are recorded in the corresponding row of the pocket-term table. For example, the GO term T1 is annotated to 8 of the closest neighbors, and the GO term T2 is annotated to 6 of the closest neighbors. The score between pocket C3 and GO terms is obtained by normalizing the row.

The correspondence scores between a pocket and GO terms are determined by its closest neighbors. The closest neighbors of a pocket are those pockets directly linked to the considered pocket. The occurrence number of each GO term in all closest neighbors is recorded in the scoring table, in which each row represents a considered pocket and each column represents a GO term. Then the scores between the considered pocket and GO terms are assigned by the frequencies of GO terms, which are the normalized occurrence numbers of GO terms, i.e. each number in the scoring table is divided by the sum of elements in the row that it is located. The scoring scheme for a pocket and GO terms is illustrated in Figure 7. We do not use any prior assumption that some pockets are related to particular GO terms. But if the relationship between some functional pockets and GO terms is confirmed by experiments, the information can be easily adopted in the scoring system.

**Figure 8:**
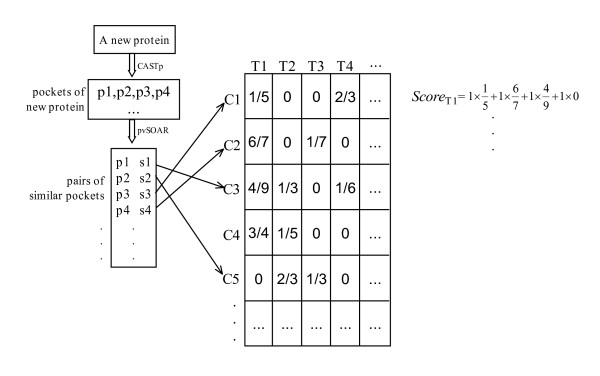
**Procedure of protein function prediction**. An example of protein scoring scheme. The target protein is queried in CASTp sever to obtain its pockets. Then the correspondence scores between pockets and GO terms are inferred as shown in Figure 5. The weighted sum of scores of all pockets with regard to a GO term is the score of the protein with regard to the GO term.

After the scores are calculated for all pockets of the target protein, the correspondence scores between the protein and GO terms are inferred from the scores of pockets. For a considered GO term, the weighted sum of the correspondence scores of all pockets in the target protein is the score of the protein with regard to the GO term. An example is illustrated in Figure 8. In this example and the current work, all pockets have the same weight value 1. In practice, if a pocket shows stronger relationship to protein functions, or the pocket is thought important to the target protein, it can be given a high weight.

### Evaluating Results of Function Prediction

The prediction performance of the proposed method is evaluated by some widely used measurements in information retrieval research, such as recall, precision and F-measure. The evaluation is usually displayed in a recall-precision graph. And the F-measure can be used as a single measure of performance of the test, which is the harmonic mean of precision and recall. Mathematically, these measurements are defined as follows.

Precision P = TP/(TP+FP)

Recall R = TP/(TP+FN)

F-measure = 2 × P × R/(P+R)

where the TP, FP and N are abbreviations of the number of true positive, number of false positive, and number of false negative respectively. The global performance is evaluated by using leave-one-out cross-validation experiments. The specific evaluation of prediction performance on each individual protein is also calculated in the similar way.

## Abbreviations

PDB: protein data bank

cRMSD: coordinate root mean square distance

oRMSD: orientation root mean square distance

CASTp: computed atlas of surface topography of proteins

pvSOAR: pocket and void surfaces of amino acid residues

GO: gene ontology

GOA: gene ontology annotation

RP: recall-precision

R & P: recall and precision

TP: true positive

FP: false positive

FN: false negative

PMID: PubMed identifier

## Authors' contributions

LYW and YW proposed the original idea, ZPL and LYW designed the details and implemented the experiments, LC and XSZ improved the methods and give valuable suggestions. All authors wrote and approved the manuscript.

## Supplementary Material

Additional file 1 Illustration examples and additional results. The illustration examples of the pocket similarity network and the similar pockets are provided. The file also includes additional statistics and prediction results. Click here for file
