# Supplementary material for: Predicting gene ontology functions from protein's regional surface structures
Source: BMC Bioinformatics. 2007 Dec 11;8:475. doi: 10.1186/1471-2105-8-475 (PMC2233648; doi:10.1186/1471-2105-8-475)
Supplement: Additional file 1 — Illustration examples and additional results. The illustration examples of the pocket similarity network and the similar pockets are provided. The file also includes additional statistics and prediction results. [file 1471-2105-8-475-S1.pdf]

Predicting gene ontology functions from protein's regional surface structures

Zhi-Ping Liu Ling-Yun Wu Yong Wang Luonan Chen Xiang-Sun Zhang

Additional file 1: Illustrative examples and additional results

A. Illustration examples

Pocket similarity network

An example of surface pocket similarity network is shown in Figure 1.

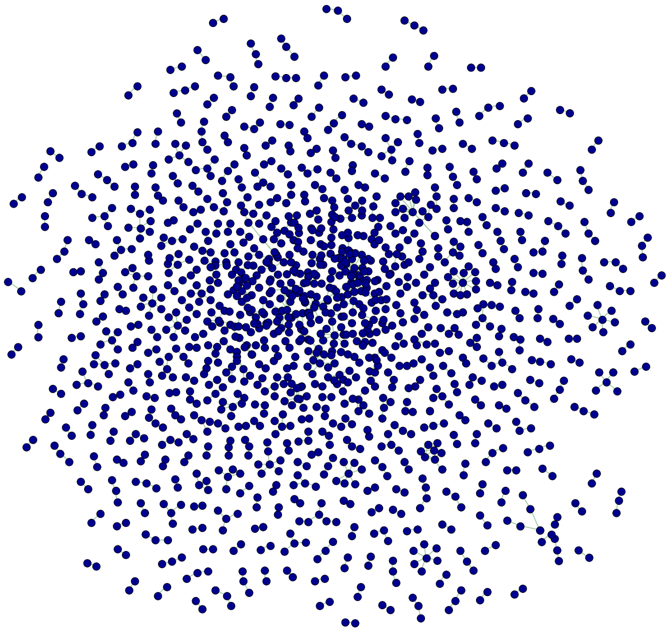

Figure 1: The partial pocket similarity network constructed by using the cRMSD p-value 10^{-1} as the threshold. From the simple network model, we can clearly detect the relationships among all pockets. This figure was prepared using the BioLayout software at: http://cgg.ebi.ac.uk/services/biolayout/.

Pocket pairs and closest neighbors

An example of pocket query results in pvSOAR database is shown in Figure 2. We also illustrate the closest neighbors of the example pocket.

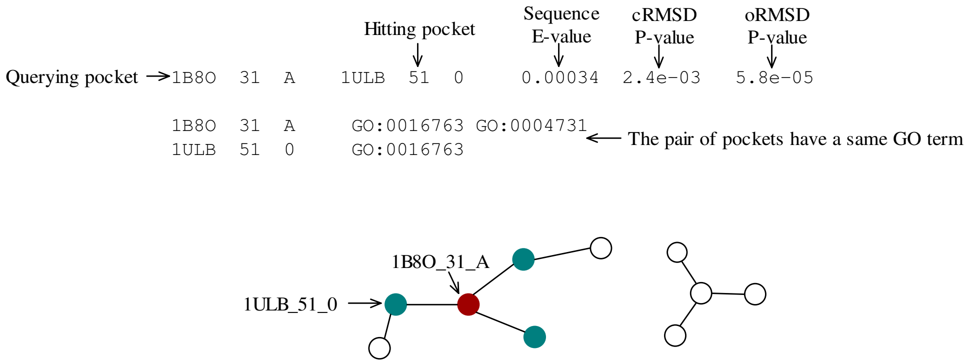

Figure 2: The querying pocket 1B8O_31_A in pvSOAR database. 1ULB_51_0 is one of the hitting pocket which satisfies the required threshold. Three similarity scores between the queried pocket and the hitting pocket are shown in the same line. The GO terms annotated to their corresponding proteins 1B8O and 1ULB are listed respectively. Since two proteins have at least one common GO term GO:0016763, the edge representing the pocket pair 1B8O_31_A and 1ULB_51_0 is a GO related edge. The closest neighborhood of pocket 1B8O_31_A is shown in the bottom of figure. The pocket 1B8O_31_A has three closest neighbors.

B. Additional statistics results

Sizes of pocket similarity networks

The sizes of pocket similarity networks constructed by using different similarity thresholds are listed in Table 1.

Table 1: The numbers of nodes and edges of the pocket similarity networks constructed by different similarity thresholds.

Threshold	1.0×10^{-1}		1.0×10^{-2}		1.0×10^{-3}		1.0×10^{-4}		1.0×10^{-5}	
	nodes	edges	nodes	edges	nodes	edges	nodes	edges	nodes	edges
Sequence E-value	3876	3178	2094	1460	1037	652	527	320	370	219
cRMSD p-value	1455	1002	831	602	711	521	655	405	634	468
oRMSD p-value	3123	2354	1634	1182	1039	757	830	618	761	567
E-value & cRMSD	1108	711	570	360	416	257	314	189	242	145

Functional similarity between pocket and its closest neighbors

We study the relationship between the pocket and the most frequent function among its closest neighbors. The statistics results are shown in Tables 2–5. The statistics are conducted on the pocket similarity networks constructed by different similarity thresholds. The 'GO Annotated' line calculates the numbers of pockets which have GO annotations and at least one closest neighbor with GO annotations. 'Similar Pockets' line records the numbers of pockets which have the most frequent function among closest neighbors. The high

percentages show the strong relationship between the pocket and the most frequent function among its closest neighbors.

Table 2: Statistics results of the functional similarity between the pocket and the most frequent function of its closest neighbors. The pocket similarity network is constructed by using the E-value of sequence similarity as the threshold.

Threshold	1.0×10^{-1}	1.0×10^{-2}	1.0×10^{-3}	1.0×10^{-4}	1.0×10^{-5}
Pockets	3876	2094	1037	527	370
GO Annotated	3008	1599	800	408	278
Similar Pockets	835	596	417	301	220
Percentage	27.76%	37.27%	52.13%	73.77%	79.14%

Table 3: Statistics results of the functional similarity between the pocket and the most frequent function of its closest neighbors. The pocket similarity network is constructed by using the p-value of structure similarity cRMSD as the threshold.

Threshold	1.0×10^{-1}	1.0×10^{-2}	1.0×10^{-3}	1.0×10^{-4}	1.0×10^{-5}
Pockets	1455	831	711	655	634
GO Annotated	1118	683	591	548	536
Similar Pockets	668	615	579	544	534
Percentage	59.75%	90.04%	97.97%	99.27%	99.63%

Table 4: Statistics results of the functional similarity between the pocket and the most frequent function of its closest neighbors. The pocket similarity network is constructed by using the p-value of structure similarity oRMSD as the threshold.

Threshold	1.0×10^{-1}	1.0×10^{-2}	1.0×10^{-3}	1.0×10^{-4}	1.0×10^{-5}
Pockets	3123	1634	1039	830	761
GO Annotated	2389	1255	833	685	646
Similar Pockets	798	716	665	645	632
Percentage	33.40%	57.05%	79.83%	94.16%	97.83%

Table 5: Statistics results of the functional similarity between the pocket and the most frequent function of its closest neighbors. The pocket similarity network is constructed by using the combination of E-value of sequence similarity and p-value of structure similarity cRMSD as the threshold.

Threshold	1.0×10^{-1}	1.0×10^{-2}	1.0×10^{-3}	1.0×10^{-4}	1.0×10^{-5}
Pockets	1108	570	416	314	242
GO Annotated	867	473	338	252	188
Similar Pockets	609	462	338	252	188
Percentage	70.24%	97.67%	100%	100%	100%

Frequent functions associated with similar pocket pairs

We study the most frequent GO functions associated with similar pocket pairs, attempting to find which kinds of GO functions are frequently shared by two proteins with similar pockets. From the statistical results

in the article, we choose cRMSD p-value 10^{-5} as the threshold to construct the pocket similarity network. The top 15 of the most frequent GO functions and their GO descriptions are shown in Table 6. Note that most of them are related to binding or catalytic activity. The full list of GO terms and their frequencies can be found on our web site.

Table 6: The top 15 of the most frequent GO functions associated with similar pocket pairs (edges) in pocket similarity network constructed by using the cRMSD p-value 10^{-5} as the threshold. There are 397 GO annotated edges, i.e. both ends with GO annotations, in total 468 edges. There are 396 edges with at least one identical GO term between two corresponding proteins. The frequency is the number of edges whose both ends have the GO term.

No.	GO term	frequency	GO description
1	GO:0005506	85	iron ion binding
2	GO:0009055	85	electron carrier activity
3	GO:0016491	48	oxidoreductase activity
4	GO:0003824	47	catalytic activity
5	GO:0004607	24	asparaginase activity
6	GO:0005524	20	ATP binding
7	GO:0003723	16	RNA binding
8	GO:0016709	16	oxidoreductase activity, acting on paired donors
9	GO:0015049	16	methane monooxygenase activity
10	GO:0030170	15	pyridoxal phosphate binding
11	GO:0008660	15	1-aminocyclopropane-1-carboxylate deaminase activity
12	GO:0004672	14	protein kinase activity
13	GO:0005164	13	tumor necrosis factor receptor binding
14	GO:0016151	13	nickel ion binding
15	GO:0004674	12	protein serine/threonine kinase activity

C. Additional prediction results

Prediction results using another scoring scheme

We also use another scoring scheme for pockets which is based on an observation that the pocket similarity networks are very sparse. We treat each connected components in the network as a similar pocket group. Then the scores of pockets are evaluated from the pockets in the same group instead of the closest neighbors. The remain procedures of learning and prediction are the same as the closest-neighbor-based method. The experiments are also performed in the pocket similarity network using cRMSD p-value 10^{-2} as threshold. The recall-precision graphs and the prediction results are shown in Figure 3 and Table 7. The results are very similar to those of the closest-neighbor-based method. One of the possible reasons is that most groups are internally densely connected, and therefore the similar pockets group are almost the same as the closest neighborhood.

Prediction results using different thresholds

We use different cRMSD p-value thresholds to construct the pocket similarity network, ranged from 10^{-3} to 10^{-5}. For each threshold, we do the same experiments as those in the article. The recall-precision graphs and prediction results are shown in Figures 4–7 and Tables 8–11. These results are very similar to the results in the article, which using cRMSD p-value 10^{-2} as the threshold.

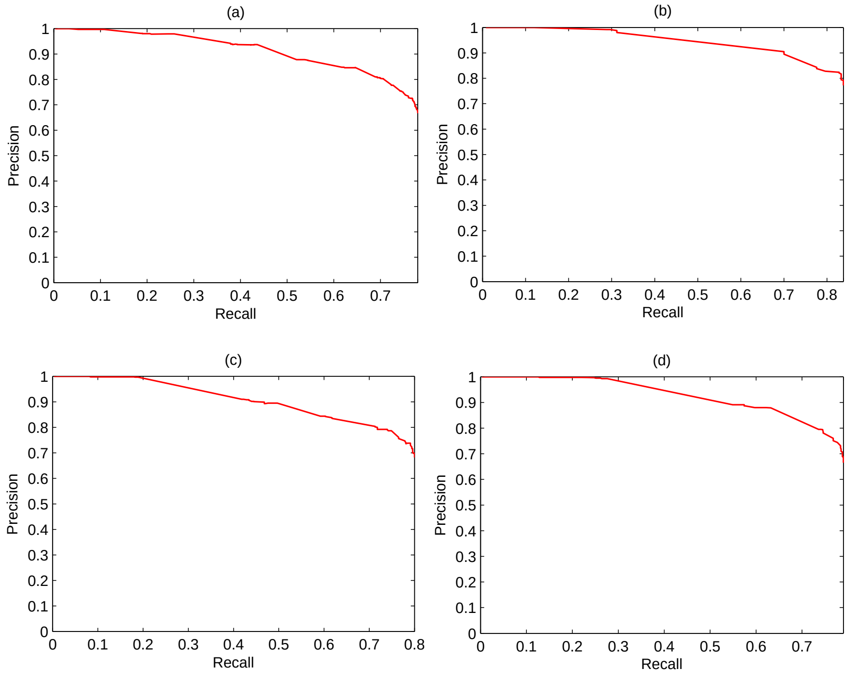

Figure 3: Recall-precision graphs when using the connected components as the similar pocket groups. (a) is the RP graph for integrated GO terms. (b), (c) and (d) are the graphs corresponding to C, F and P ontology, respectively.

Table 7: Separated prediction results by using the connected components as the similar pocket groups.

Ontology	Integrated	C	F	P
Maximum F-measure	0.752	0.826	0.768	0.768
Recall-precision	(0.780,0.667)	(0.838,0.772)	(0.800,0.682)	(0.790,0.665)
Number of proteins	320	100	294	280
Predicted proteins	271	89	251	235
Not predicted	49	11	43	45
Proteins with recall 100%	219	79	213	206
Proteins with precision 100%	165	71	160	155
Proteins with R & P 100%	148	66	148	143

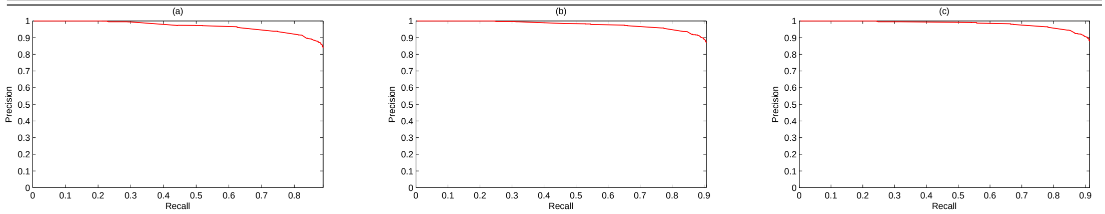

(a) 10^{-3} (b) 10^{-4} (c) 10^{-5}

Figure 4: Recall-precision graphs for prediction results in the pocket similarity network by using different cRMSD p-values as thresholds.

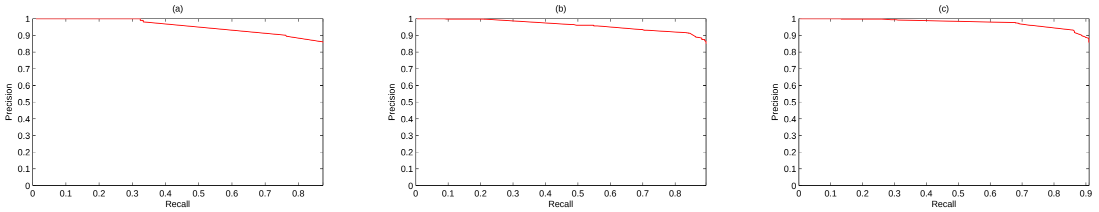

(a) cellular component (b) molecular function (c) biological process

Figure 5: Recall-precision graphs for separated prediction results in the pocket similarity network by using cRMSD p-value 10^{-3} as the threshold.

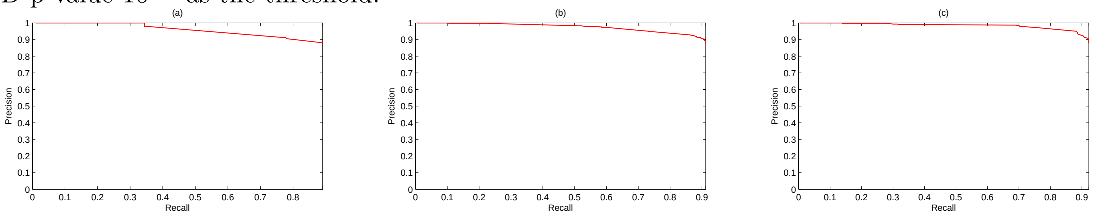

(a) cellular component (b) molecular function (c) biological process

Figure 6: Recall-precision graphs for separated prediction results in the pocket similarity network by using cRMSD p-value 10^{-4} as the threshold.

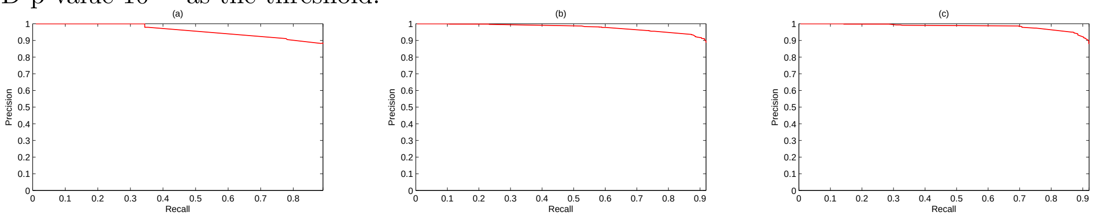

(a) cellular component (b) molecular function (c) biological process

Figure 7: Recall-precision graphs for separated prediction results in the pocket similarity network by using cRMSD p-value 10^{-5} as the threshold.

Table 8: Prediction results in the pocket similarity network by using different cRMSD p-values as thresholds.

Threshold	10^{-3}	10^{-4}	10^{-5}
Maximum F-measure	0.878	0.903	0.910
Recall-precision	(0.888, 0.839)	(0.907, 0.869)	(0.913, 0.877)
Number of proteins	265	242	237
Predicted proteins	257	238	235
Not predicted	8	4	2
Proteins with recall 100%	206	193	190
Proteins with precision 100%	185	180	178
Proteins with R & P 100%	160	155	153

Table 9: Separated prediction results in the pocket similarity network by using cRMSD p-value 10^{-3} as the threshold.

Ontology	C	F	P
Maximum F-measure	0.868	0.883	0.896
Recall-precision	(0.874,0.857)	(0.895,0.850)	(0.910,0.858)
Number of proteins	86	249	229
Predicted proteins	79	241	224
Not predicted	7	8	5
Proteins with recall 100%	72	198	192
Proteins with precision 100%	70	179	172
Proteins with R & P 100%	67	159	153

Table 10: Separated prediction results in the pocket similarity network by using cRMSD p-value 10^{-4} as the threshold.

Ontology	C	F	P
Maximum F-measure	0.888	0.905	0.915
Recall-precision	(0.891,0.879)	(0.912,0.877)	(0.922,0.881)
Number of proteins	79	228	213
Predicted proteins	73	223	211
Not predicted	6	5	2
Proteins with recall 100%	68	186	182
Proteins with precision 100%	67	175	169
Proteins with R & P 100%	64	154	149

Table 11: Separated prediction results in the pocket similarity network by using cRMSD p-value 10^{-5} as the threshold.

Ontology	C	F	P
Maximum F-measure	0.888	0.912	0.912
Recall-precision	(0.891,0.879)	(0.919,0.886)	(0.920,0.879)
Number of proteins	79	223	210
Predicted proteins	73	220	208
Not predicted	6	3	2
Proteins with recall 100%	68	183	179
Proteins with precision 100%	67	173	166
Proteins with R & P 100%	64	152	146

D. Prediction results by the protein similarity network

We construct the corresponding protein similarity networks in the similar way of constructing the pocket similarity networks. The global structure similarity between the proteins is measured by CE (combinatorial extension, Shindyalov and Bourne, Protein Engineering 1998). We do the similar testing and compare the results with those which we have achieved in the pocket similarity networks. The prediction results on the protein similarity networks constructed by different CE Z-Scores 3.8, 4.8, 5.8 are shown in Figure 8 (CE recommended Z-Score 3.8 as the threshold to filter out structure similarities). The details of the prediction are listed in Table 12.

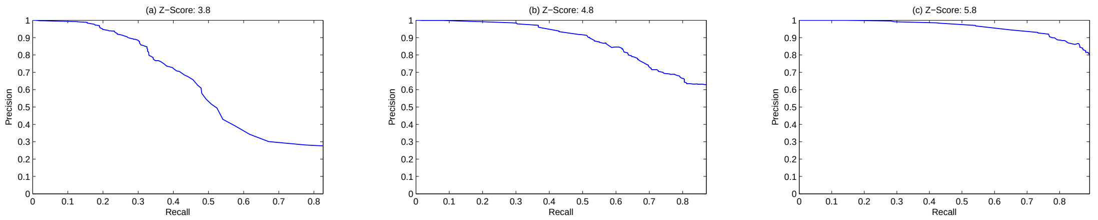

(a) Z-Score: 3.8 (b) Z-Score: 4.8 (c) Z-Score: 5.8

Figure 8: Recall-precision graphs for the protein similarity networks constructed by different thresholds. (a), (b) and (c) represent the RP curves of predictions for CE Z-Score 3.8, 4.8 and 5.8 respectively.

Table 12: Prediction results by the protein similarity networks using different CE Z-Scores as thresholds.

Threshold	3.8	4.8	5.8
Maximum F-measure	0.538	0.730	0.862
Recall-precision	(0.826, 0.276)	(0.871, 0.628)	(0.892, 0.804)
Number of proteins	325	280	235
Predicted proteins	301	268	230
Not predicted	23	12	5
Proteins with recall 100%	226	213	182
Proteins with precision 100%	47	115	146
Proteins with R & P 100%	44	97	127

We compare the results of pocket similarity networks by thresholds p-value 10^{-2}, 10^{-3} with those of protein similarity networks by Z-Scores 4.8, 5.8 in an all-against-all manner. The detail of comparison can be found in Table 13. The comparison of RP curves can be found in Figure 9 in addition to the two RP curves comparison graphs shown in the text.

In Table 13, "ProtNum" means the number of proteins in different similarity networks. "Common" represents the common proteins in the two kinds of networks. "Max F" is the maximum F-measure. "R-P" represents recall-precision. "R 100%" and "P 100%" mean that the number of proteins can be predicted with recall value 1 and with precision value 1 respectively. "R & P 100%" represents the proteins which can be prediction with both recall value 1 and precision value 1.

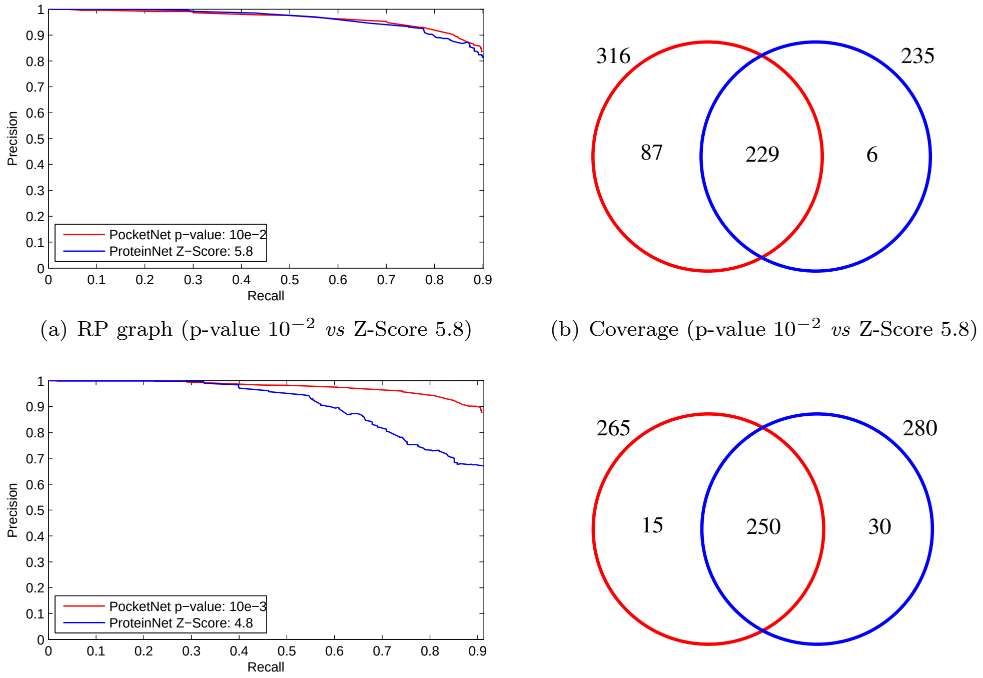

(a) RP graph (p-value 10^{-2} *vs* Z-Score 5.8)

(b) Coverage (p-value 10^{-2} *vs* Z-Score 5.8)

(c) RP graph (p-value 10^{-3} *vs* Z-Score 4.8)

(d) Coverage (p-value 10^{-3} *vs* Z-Score 4.8)

Figure 9: Comparison of recall-precision graphs and coverage between the results by the pocket similarity network *versus* those by protein similarity network.

Table 13: Comparison of the prediction results by different similarity networks.

Net	Threshold	ProtNum	Common	Max F	R-P	R 100%	P 100%	R & P 100%
Pocket	10^{-2}	316	273	0.849	(0.867, 0.805)	211	178	155
Protein	4.8	280		0.736	(0.878, 0.633)	212	115	97
Pocket	10^{-3}	265	219	0.908	(0.916, 0.888)	177	164	141
Protein	5.8	235		0.889	(0.918, 0.832)	177	144	125
Pocket	10^{-2}	316	229	0.875	(0.899 0.834)	183	154	133
Protein	5.8	235		0.871	(0.902, 0.813)	182	146	127
Pocket	10^{-3}	265	250	0.901	(0.909, 0.876)	201	184	159
Protein	4.8	280		0.774	(0.913, 0.671)	203	113	95

E. An example of two proteins with similar pockets

The following Figure 10 is an example of two proteins with similar pockets. Figure 10 (a) is the global folding of the two proteins. They have one similar pocket. The positions where the two pockets locate on the protein surfaces are also shown. Part (b) is the visualization of the two similar pockets. (c) is the sequences of the two proteins. The red characters are the amino acid residues of the two similar pockets. They locate non-consecutively on the protein sequences individually. The GO annotations (EBI, http://www.ebi.ac.uk/goa/) to the proteins are also listed. The two proteins have some common GO functions. When we align the two protein sequences by EMBOSS (EBI, http://www.ebi.ac.uk/emboss/), the sequence identity of the two proteins is 7.9%, the sequence similarity is 11.7%. The results of structure alignment is 4.9Å(RMSD) and 1.2(Z-Score) by CE (UCSD, http://cl.sdsc.edu/). From the sequence and structure alignment, we can find that they are with both low sequence similarity and low globally structure similarity. (d) The concatenated pocket sequences show that the two pockets have high sequence similarity. The cRMSD of the pvSOAR (UIC, http://pvsoar.bioengr.uic.edu/) structure alignment between the two pockets is 0.414Å, the p-value is 4.054×10^{-8}. Figures of protein structures and pocket architectures are prepared by the Jmol from the CASTp database (UIC, http://cast.engr.uic.edu).

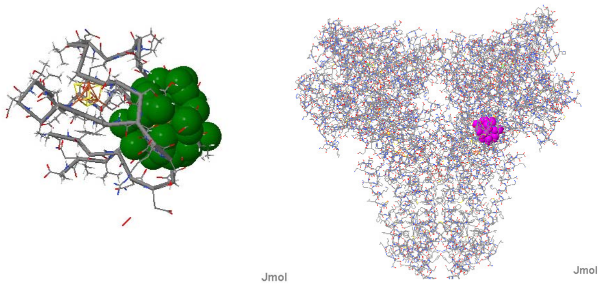

Protein: 2fdn   Protein: 1qla

(a)

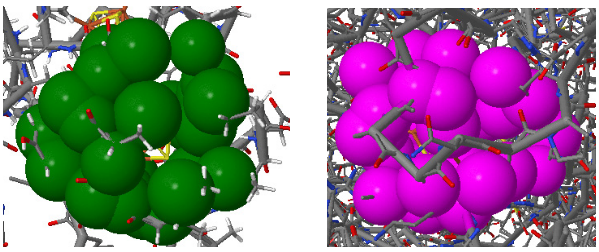

Pocket: 2fdn_4_0   Pocket: 1qla_303_E

(b)

>2fdn: GO:0005506; GO:0009055; GO:0006118
AYVINEACISCGACEPECPVNAISSGDDRYVIDADTCIDCGACAGVCPVDAPVQA

>1qla_E: GO:0005506; GO:0009055; GO:0016491;GO:0006118; GO:0006099; GO:0016020
MGRMLTIRVFKYDPQSAVSKPHFQEYKIEEAPSMTIFIVLNMIRETYDPDLNFDFVCRAG
ICGSCGMMINGRPSLACRTLTKDFEDGVITLLPLPAFKLIKDLSVDTGNWFNGMSQRVES
WIHAQKEHDISKLEERIEPEVAQEVFELDRCIECGCCIAACGTKIMREDFVGAAGLNRVV
RFMIDPHDERTDEDYYELIGDDDGVFGCMTLLACHDVCPKNLPLQSKIAYLRRKMVSVN

EMBOSS: Identity = 19/239(7.9%), Similarity = 28/239(11.7%)
CE: RMSD = 4.9Å, Z-Score = 1.2

(c)

2fdn_4_0: ICISCGACYVCPVAP
1qla_303_E: FCIECGCCANCPKLL

pvSOAR: cRMSD = 0.414Å, p-value = 4.054e-08

(d)

Figure 10: A pair of proteins with similar pockets. (a) is the structures of two proteins and (b) is the structures of the pockets. The GO annotations of the proteins and the sequence alignment and structure alignment are provided in (c). (d) show the comparison between two pockets.

From the example we can find that the two proteins with similar function, but they have both low sequence similarity and structure similarity. If we predict protein functions from the sequence and/or global structure similarity, it would be difficult to get the correct predictions. However, we can find that the pockets located on the different protein surfaces have the sequence and structure similarity. When we predict function by the method proposed in the paper, we can get the correct annotations. The detailed descriptions of the GO terms and the common annotations of the two proteins are shown in Table 14. The depth is the level that the GO term located in the GO hierarchy tree, when the GO term occurs in different branches, the mean level is used.

Table 14: The detailed description to the GO terms which are annotated to protein 2fdn and 1qla_E. The italic GO terms are the common GO annotations between the two proteins.

GO term	Ontology	Probability	Depth	description
GO:0005506	F	0.064	6	iron ion binding
GO:0009055	F	0.071	4	electron carrier activity
GO:0006118	P	0.139	4	electron transport
GO:0016491	F	0.179	3	oxidoreductase activity
GO:0006099	P	0.007	7	tricarboxylic acid cycle
GO:0016020	C	0.363	4	membrane

The example also gives us implications that the functionally important pockets on protein surface would have important applications in functional genomics and in bioengineering. The similar pockets in this example are the ion binding sites (which can be identified from the Swissprot database). The functional residues lie in the primary sequence discontinuously. However, when these residues fold into 3D shape, they would constitute a binding site to perform concrete function. If we can detect the biochemical features of pockets and identified these pockets as functional motifs, these pockets would be the functional templates. The template-based method to predict function is an important future direction.

F. Semantic measure of the functional similarity

The proteins are often annotated with several GO terms simultaneously. The semantic similarity can be used to compare the functional similarity between two proteins along the edges in the pocket similarity network, instead of the simple method by considering the common GO terms. Two GO term sets of the proteins can be compared in three ontologies individually. The relevance semantic similarity score of each edge in the pocket similarity networks constructed by different cRMSD p-value thresholds, ranged from 10^{-1} to 10^{-5}, is calculated by the method proposed in the literature (Schlicker A. et al., BMC Bioinformatics, 2006). The scores in three ontologies are calculated independently and the semantic similarity values range between 0 and 1. The distributions of the semantic similarity scores in different pocket networks are illustrated in Figure 11, i.e. the percentage of GO annotated edges in each semantic similarity score interval. The figures show that the pocket similarity (represented by the edges in the pocket similarity networks) is closely related to the semantic similarity.

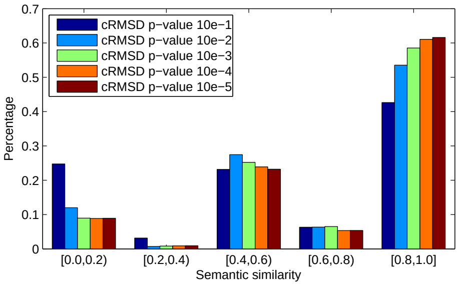

(a) cellular component

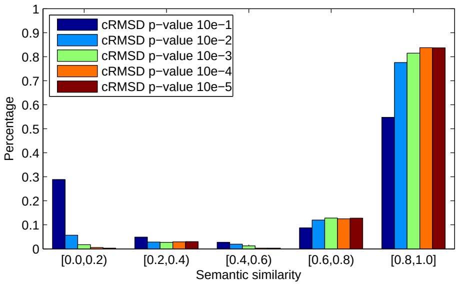

(b) molecular function

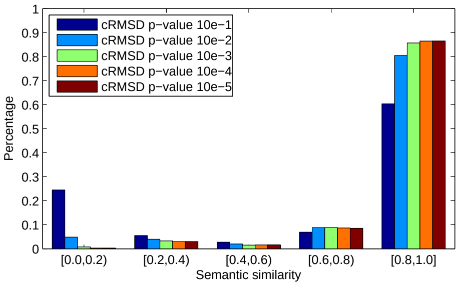

(c) biological process

Figure 11: The distribution of the semantic similarity scores of the GO annotated edges in the pocket similarity networks constructed by different cRMSD p-values.

G. Influence of GO relevance information

The GO organizes the terms as directed acyclic graphs (DAG), where the child term is more specific and informative than its ancestors. To analyze the influence of the unspecific GO terms in the proposed prediction method, we use the GO term probability and GO depth level to select informative GO terms.

The GO term probability

Table 15 shows the detailed prediction results by using the GO term probability thresholds 0.05, 0.01 and 0.005 respectively.

Table 15: Prediction results in the pocket similarity network (constructed by cRMSD p-value 10^{-2}) by using the GO term probability to select informative GO terms.

GO frequency threshold	0.05	0.01	0.005
Maximum F-measure	0.742	0.730	0.748
Recall-precision	(0.757, 0.708)	(0.734, 0.707)	(0.750,0.714)
Number of proteins	292	215	158
Predicted proteins	239	169	121
Not predicted	53	46	37
Proteins with recall 100%	201	150	116
Proteins with precision 100%	174	139	105
Proteins with R & P 100%	154	125	102

For the selected informative GO terms with probability less than 0.01, we also compare the prediction results by the pocket similarity network (constructed by pvSOAR cRMSD p-value 10^{-2}) and by protein similarity network (constructed by CE Z-score 4.8). The recall-precision graph in their common predicted proteins and the coverage of the predicted proteins are shown in Figure 12. Table 16 records the detailed values of prediction in the common proteins.

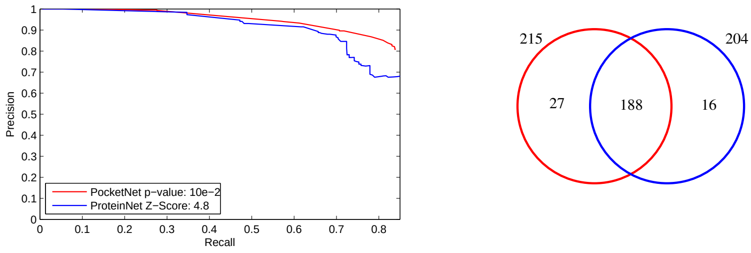

(a) (b)

Figure 12: Recall-precision graph and coverage of the results by the pocket similarity network and by protein similarity network for the GO terms with probability less than 0.01.

Table 16: Details of the prediction results by the pocket similarity network and by protein similarity network for the GO terms with probability less than 0.01

Net	Threshold	ProtNum	Common	Max F	R-P	R 100%	P 100%	R & P 100%
Pocket	10^{-2}	215	188	0.831	(0.839, 0.809)	150	139	125
Protein	4.8	204		0.78	(0.850, 0.680)	152	104	94

The GO depth level

An alternative simple way to select informative GO terms is the depth level of GO terms. Figure 13 shows the distribution of GO terms with different depth levels. When a GO term belongs to several branches in the hierarchical tree and have different depth level labels, the mean value is used.

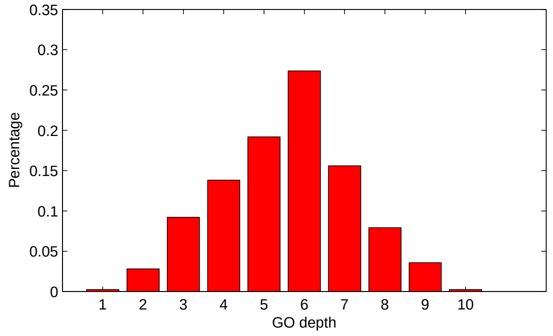

Figure 13: The distribution of GO terms with different depth levels in the dataset.

Table 17 shows the detailed prediction results by using the GO depth level thresholds 5, 6, 7 and 8 respectively.

Table 17: Prediction results in the pocket similarity network (constructed by cRMSD p-values 10^{-2}) by using the GO depth level to select informative GO terms.

GO depth	≥ 5	≥ 6	≥ 7	≥ 8
Maximum F-measure	0.788	0.822	0.778	0.800
Recall-precision	(0.806, 0.733)	(0.839, 0.757)	(0.781, 0.768)	(0.800, 0.778)
Number of proteins	278	215	113	45
Predicted proteins	242	193	93	36
Not predicted	36	22	20	9
Proteins with recall 100%	205	165	82	36
Proteins with precision 100%	177	142	80	34
Proteins with R & P 100%	155	127	72	34

We also compare the prediction results by the pocket similarity network (constructed by pvSOAR cRMSD p-value 10^{-2}) and by the protein similarity network (constructed by CE Z-Score 4.8) for the GO terms with depth level ≥ 5. The recall-precision graph in their common predicted proteins and the coverage of the predicted proteins are shown in Figure 14. Table 18 records the detailed values of the prediction results in the common proteins.

Table 18: Details of the prediction results by the pocket similarity network and by protein similarity network for the GO terms with depth level ≥ 5 .

Net	Threshold	ProtNum	Common	Max F	R-P	R 100%	P 100%	R & P 100%
Pocket	10^{-2}	278	246	0.862	(0.880, 0.818)	200	176	154
Protein	4.8	256		0.768	(0.887, 0.645)	203	112	102

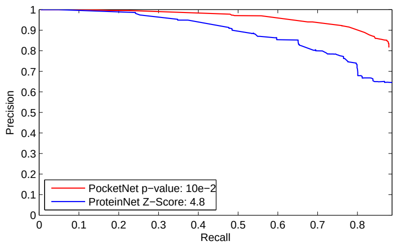

(a)

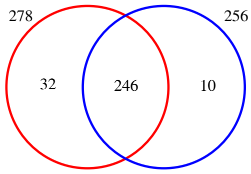

(b)

Figure 14: Recall-precision graph and coverage of the results by the pocket similarity network and by protein similarity network for the GO terms with depth level ≥ 5.
